# Graphene-Confined Silicon and a Li_6.5_La_3_Zr_1.5_Ta_0.5_O_12_-Reinforced Plasticized Poly(ethylene oxide) Electrolyte Enable Contact-Stable Solid-State Lithium Batteries

**DOI:** 10.3390/polym18192374

**Published:** 2026-09-29

**Authors:** Xianzheng Liu, Nashrah Hani Jamadon, Jiayi Li, Xiaoxi Liu, Wenbo Jia, Rongji Tang, Liancheng Zheng, Zhenhua Liu, Dongpo Wei

**Affiliations:** 1 College of Mechanical Engineering, Shandong Huayu University of Technology, Dezhou 253034, China; 2 Department of Mechanical and Manufacturing Engineering, Faculty of Engineering and Built Environment, Universiti Kebangsaan Malaysia, Bangi 43600, Selangor, Malaysia; 3 Department of Electrical, Electronic and Systems Engineering, Faculty of Engineering and Built Environment, Universiti Kebangsaan Malaysia, Bangi 43600, Selangor, Malaysia

**Keywords:** silicon anode, reduced graphene oxide, composite polymer electrolyte, LLZTO, solid-state battery, chemo-mechanical interface

## Abstract

Silicon is a promising anode for solid-state lithium batteries because of its high theoretical capacity, but large lithiation-induced volume changes and unstable electrode/electrolyte contact remain major challenges. Here, a contact-adaptive solid-state silicon (Si) architecture is developed by coupling an electrostatically assembled silicon/reduced graphene oxide (Si@rGO) nanosheet anode with a poly(ethylene oxide) (PEO)–lithium bis(trifluoromethanesulfonyl)imide (LiTFSI)–ethylene carbonate (EC) electrolyte reinforced with 10 wt% Ta-doped Li_6.5_La_3_Zr_1.5_Ta_0.5_O_12_ (LLZTO), hereafter denoted PEC-T. Trace EC promotes Li-salt solvation and PEO plasticization, while LLZTO suppresses polymer crystallization and reinforces ion transport. PEC-T shows a reduced melting temperature from 66.7 to 51.4 °C, improved tensile strength and elongation, an ionic conductivity of 3.0 × 10^−4^ S cm^−1^ at 30 °C, a Li^+^ transference number of 0.57, and an oxidative stability limit of ~4.5 V. It also supports stable Li plating/stripping for 600 h at 0.1 mA cm^−2^. Meanwhile, the rGO framework mitigates irreversible Si thickness evolution and preserves interfacial integrity. Consequently, Si@rGO/PEC-T/Li batteries deliver strong rate capability and maintain 1.8 Ah g^−1^ after 250 cycles at 1 C, demonstrating a coupled chemo-mechanical strategy for stable solid-state silicon batteries.

## 1. Introduction

The transition from liquid-electrolyte lithium-ion batteries to solid-state configurations is motivated by the need to simultaneously raise energy density and reduce flammability-associated safety risk [1,2,3]. However, replacing a liquid with a solid does not simply remove the electrolyte as a failure source; it transfers much of the electrochemical burden to buried solid–solid interfaces, where ion transport, electrochemical stability, and stress evolution become strongly coupled [4,5]. This coupling is particularly severe for silicon. Si can alloy with large amounts of lithium and offers a theoretical capacity far beyond graphite, but the associated structural reorganization and large volume change repeatedly perturb electrical pathways and the electrode/electrolyte contact network [6,7,8]. In liquid batteries, continuous interphase rupture and reformation consume electrolyte and cyclable lithium; in solid-state batteries, the additional challenge is the progressive loss of physical contact as the active material contracts and expands against a mechanically dissimilar electrolyte [9,10].

Recent studies have therefore shifted the design target from simply maximizing the intrinsic capacity of Si toward regulating its structural and interfacial evolution [11,12,13]. A recent comprehensive review by Toki et al. emphasized that the practical limitations of Si-based anodes arise not only from the large lithiation-induced volume change, but also from repeated interphase reconstruction, electrical isolation, low initial Coulombic efficiency, and insufficient electrode-level stability, making coordinated material, binder, electrolyte, and interface engineering increasingly important [14]. Nanostructuring, carbon confinement, alloying, and engineered composite electrodes can create internal free volume, maintain electronic percolation, and redistribute reaction-induced stress [15,16,17]. Reduced graphene oxide (rGO) is particularly attractive because its high aspect ratio can generate a continuous conductive scaffold while its flexible sheets accommodate local displacement [18,19]. Recent rGO/Si architectures further demonstrate this concept. Cheng et al. constructed a sandwich-like rGO/Si/rGO structure in which Si particles were confined between rGO layers, improving structural accommodation and electrochemical stability [20]. More recently, Tan et al. demonstrated that binder-free rGO–Si electrodes with controlled Si content exhibit composition-dependent interfacial kinetics and cycling behavior, highlighting the importance of simultaneously optimizing the conductive framework and Si fraction [21]. These studies support the use of rGO not merely as a conductive additive, but as a mechanically adaptive interfacial scaffold for Si-based electrodes. The importance of interface regulation becomes even more pronounced when Si is transferred from liquid-electrolyte cells to solid-state configurations. In liquid cells, the electrolyte can continuously wet newly generated Si surfaces, whereas in solid-state batteries repeated Si expansion and contraction directly perturb solid–solid contact, leading to coupled electrochemical and mechanical degradation. Recent analyses of Si-based solid-state batteries have therefore identified interfacial contact loss, local stress accumulation, reaction heterogeneity, and unstable interphase formation as mutually coupled failure modes rather than independent processes. Liang et al. recently summarized how polymer, oxide, sulfide, halide, and composite solid electrolytes interact differently with Si anodes and emphasized that successful Si-based solid-state batteries require simultaneous regulation of electrochemical compatibility and mechanical contact [22]. Similarly, Su et al. highlighted that chemo-mechanical degradation at the Si/solid-electrolyte interface links microscopic interfacial evolution to macroscopic capacity decay and argued for electrolyte structures that combine ionic transport with mechanical compliance [23]. These considerations motivate an electrode–electrolyte co-design strategy rather than optimization of either component in isolation.

Poly(ethylene oxide) (PEO)-based solid polymer electrolytes provide a complementary set of attributes. Their softness, processability, and strong solvating interaction with lithium salts enable substantially better conformability than dense ceramic electrolytes [24,25]. The central limitation is that Li^+^ transport in PEO is linked to local segmental motion, so the high crystallinity of PEO near ambient temperature suppresses conductivity and amplifies concentration polarization [26,27]. Incorporating inorganic garnet fillers such as Ta-doped lithium lanthanum zirconium oxide (LLZTO; nominal Li_6.5_La_3_Zr_1.5_Ta_0.5_O_12_) can disrupt chain packing, provide Lewis acid–base interactions at the polymer/ceramic boundary, increase mechanical robustness, and, in favorable microstructures, create additional low-barrier pathways for Li^+^ migration [28]. A 10 wt% LLZTO loading has previously emerged as a useful regime for balancing dispersion, polymer continuity, and mechanical reinforcement in PEO–lithium bis(trifluoromethanesulfonyl)imide (LiTFSI) systems [29]. More recent work further emphasizes that particle size, dopant chemistry, and polymer/LLZTO interfacial bonding can be as important as the nominal filler content itself [30].

Plasticization offers another route to reduce the kinetic penalty associated with PEO crystallinity [31]. In the present formulation, a deliberately small amount of ethylene carbonate (EC) is introduced into PEO-LiTFSI. EC can coordinate Li^+^, weaken ion pairing, and increase local chain mobility without converting the membrane into a liquid-rich gel [32]. This function is conceptually distinct from that of LLZTO: EC modifies local solvation and segmental dynamics, whereas LLZTO simultaneously perturbs crystallization and stiffens the polymer matrix. Combining these effects is therefore expected to generate a membrane that is both more conductive and mechanically better able to maintain contact with a breathing Si electrode. More broadly, recent studies in other high-volume-change battery chemistries also show that electrode stabilization cannot be achieved solely by modifying the active material. For example, Ovc-Okene et al. demonstrated in Li–S batteries that combining physical confinement with polymer-mediated interphase regulation can simultaneously improve ionic transport and preserve reaction-interface stability [33]. Although the underlying electrode chemistry differs from Si, this result illustrates a transferable design principle: structural confinement and electrolyte/interphase engineering can act cooperatively to stabilize an evolving electrode interface.

**Figure 1:**
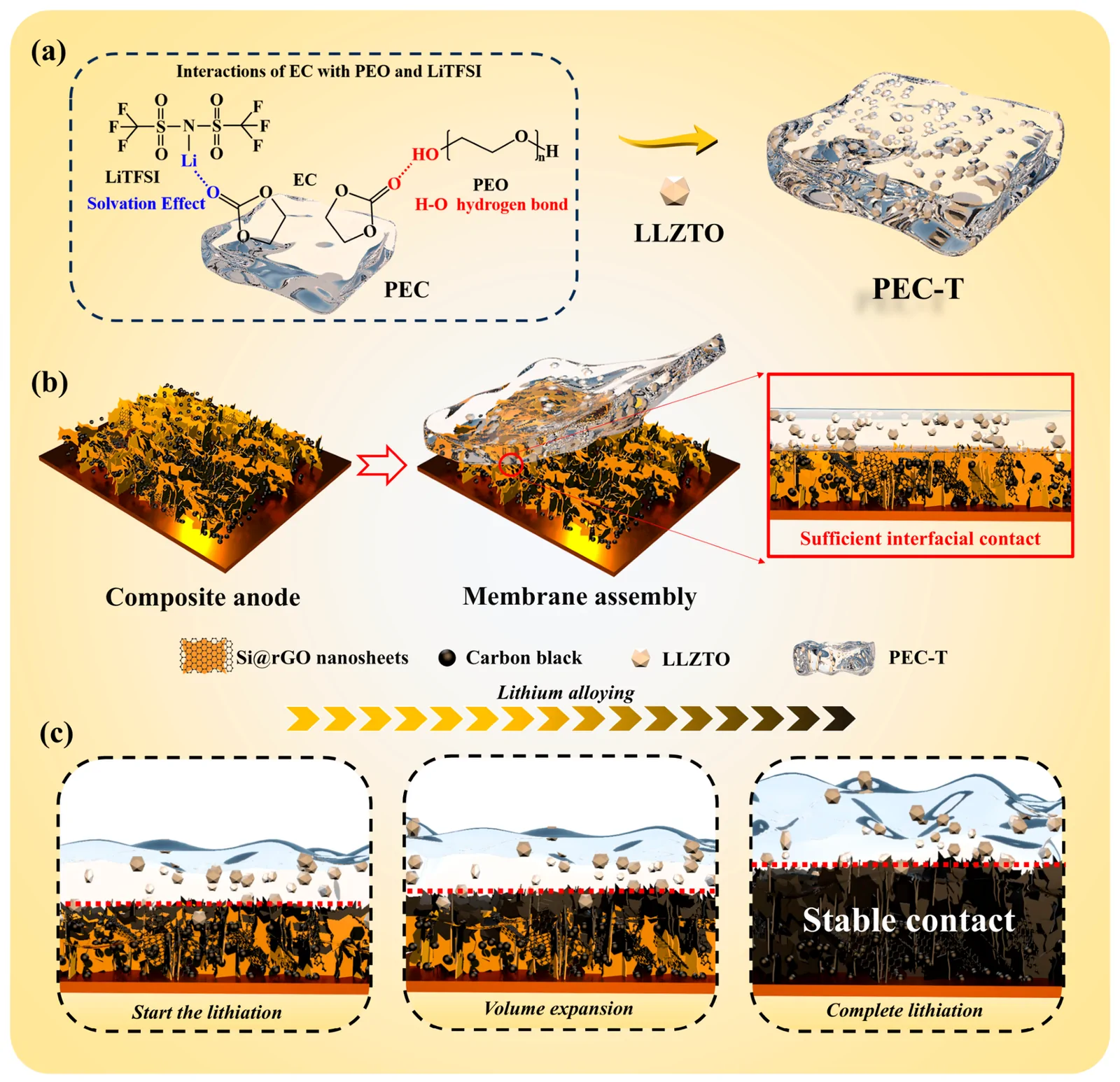
Design of the Si@rGO/PEC-T interface. (**a**) EC-assisted PEO-LiTFSI coordination and LLZTO reinforcement. (**b**) Conformal membrane/electrode contact. (**c**) Proposed preservation of ion-conducting contact during Si lithiation and expansion.

Here, we integrate these two design axes into a contact-adaptive Si@rGO/PEC-T solid-state architecture. As schematically illustrated in Figure 1, Si nanosheets recovered from high-temperature-pretreated photovoltaic cutting-Si waste are functionalized with 3-aminopropyltriethoxysilane (APTES) and electrostatically assembled with graphene oxide, followed by thermal reduction to form a conductive and mechanically compliant Si@rGO framework. In parallel, a PEO–LiTFSI–EC matrix is reinforced with 10 wt% Ta-doped LLZTO to form PEC-T. Within the electrolyte, trace EC regulates Li^+^ coordination and promotes local polymer-chain mobility, whereas LLZTO disrupts crystalline PEO domains while providing mechanical reinforcement and additional ion-transport functionality (Figure 1a). More importantly, the two components are designed to act cooperatively at the buried Si/electrolyte interface. The flexible rGO framework accommodates the dimensional evolution of Si, while the compliant PEC-T membrane conforms to the evolving electrode topography and preserves continuous ionic contact during lithiation (Figure 1b,c). Rather than attempting to eliminate Si volume change, this architecture seeks to maintain coupled electronic and ionic pathways throughout repeated expansion and contraction. By correlating structural evolution, thermal and mechanical properties, ion transport, interfacial stability, and long-term electrochemical behavior, this work establishes a coupled electrode–electrolyte strategy for stabilizing high-capacity Si anodes in solid-state batteries.

## 2. Experimental Section

### 2.1. Materials

Natural graphite powder, sodium nitrate (NaNO_3_), potassium permanganate (KMnO_4_), concentrated sulfuric acid (H_2_SO_4_), hydrogen peroxide (H_2_O_2_, 28 wt%), hydrochloric acid (HCl), 3-aminopropyltriethoxysilane (APTES), ethanol, poly(ethylene oxide) (PEO, Mw ≈ 600,000), lithium bis(trifluoromethanesulfonyl)imide (LiTFSI, battery grade), ethylene carbonate (EC, battery grade), anhydrous acetonitrile, Super P conductive carbon and sodium carboxymethyl cellulose (CMC) were used as received. Commercial natural flake graphite powder (Sigma-Aldrich, St. Louis, MO, USA; product No. 808067; 99% carbon basis; −325 mesh, with ≥99% of particles below 44 μm) was used as the precursor for graphene oxide synthesis without further purification or sieving. High-temperature-pretreated photovoltaic wafer-cutting silicon waste powder was used as the Si precursor. SEM observation (Figure S1) reveals an irregular flake-like and fractured morphology. In the representative SEM field, discernible primary fragments are mainly ~0.5–3 μm in apparent lateral dimension, with occasional larger or overlapping features approaching ~5 μm. These values represent SEM-estimated apparent dimensions rather than a laser-diffraction D50. Bulk compositional analysis showed that Si was the dominant component of the high-temperature-pretreated photovoltaic wafer-cutting waste, together with residual oxygen, carbon, and minor metallic impurities. The detailed elemental composition is summarized in Table S1 [34]. Ta-doped garnet LLZTO powder (nominal Li_6.5_La_3_Zr_1.5_Ta_0.5_O_12_; submicron grade) was used as the ceramic filler. PEO, LiTFSI and LLZTO were vacuum-dried before electrolyte preparation. Unless otherwise noted, electrolyte handling and battery assembly were conducted in an Ar-filled glovebox with O_2_ and H_2_O levels below 0.1 ppm.

### 2.2. Preparation of Graphene Oxide

Graphene oxide was synthesized using a modified Hummers-type oxidation route following the supplied experimental protocol. Briefly, 300 mL of anhydrous concentrated H_2_SO_4_ was cooled to approximately 5 °C under continuous stirring. Graphite powder (10 g) was slowly added and dispersed for 0.5 h, after which NaNO_3_ (5 g) was introduced gradually and the suspension was stirred for another 0.5 h. KMnO_4_ (45 g) was then added slowly while maintaining efficient external cooling, and the oxidation was continued for 9 h. After oxidation, 300 mL of deionized water was introduced very slowly to control the strongly exothermic dilution step. After the temperature had decreased, 30 mL of H_2_O_2_ (28 wt%) was added dropwise. The suspension was allowed to stand and separate; the solid-rich lower fraction was collected and washed with 400 mL of 5 wt% HCl no fewer than four times. The oxidized product was subsequently washed with deionized water until neutral and freeze-dried to obtain GO powder.

### 2.3. Preparation of Si@rGO

Si@rGO was prepared by APTES-assisted electrostatic assembly using the supplied experimental conditions. Photovoltaic-waste Si powder (200 mg) was dispersed in 100 mL ethanol and ultrasonicated for 0.5 h. APTES (1 mL) was added and the dispersion was ultrasonicated for a further 0.5 h to introduce amino-containing surface groups. The modified Si was collected by filtration, washed, and redispersed by ultrasonication in 100 mL ethanol. A GO suspension (200 mL, 1 mg mL^−1^) was then added, and the mixture was magnetically stirred for 6 h to drive electrostatic assembly between positively functionalized Si and negatively charged GO. The resulting Si/GO composite was collected and annealed under flowing Ar at 800 °C for 3 h (heating rate 5 °C min^−1^) to reduce GO to rGO, yielding the Si@rGO composite.

### 2.4. Preparation of PEC and PEC-T Electrolyte Membranes

The electrolyte formulation was designed around a PEO-continuous matrix with a deliberately small EC content. PEO (1.00 g) and LiTFSI (0.40 g; EO:Li ≈ 16:1) were dissolved in 12 mL anhydrous acetonitrile and stirred at 25 °C for 4 h. EC (0.05 g, 5 wt% relative to PEO) was then introduced and the solution was stirred at 60 °C for 2 h to promote homogeneous salt coordination and plasticization. This PEO-LiTFSI-EC formulation is denoted PEC. For PEC-T, LLZTO (0.10 g, corresponding to an LLZTO/PEO mass ratio of 10/100) was separately dispersed in 3 mL anhydrous acetonitrile by ultrasonication for 30 min and added slowly to the PEC solution. The precursor was stirred for an additional 6 h at 60 °C. The homogeneous slurry was cast into a PTFE mold, allowed to stand at ambient temperature for 6 h to minimize bubble formation, and vacuum-dried at 60 °C for 24 h. Free-standing membranes were transferred directly to the glovebox and punched into 16 mm disks. The final PEC-T membrane thickness was approximately 80 μm. PEC control membranes were prepared identically but without LLZTO.

### 2.5. Electrode Preparation and Battery Assembly

Si@rGO electrodes were prepared by mixing Si@rGO, Super P and CMC at a mass ratio of 8:1:1 in deionized water. The slurry was stirred until homogeneous, coated onto Cu foil, and vacuum-dried at 80 °C for 24 h. Bare-Si control electrodes were fabricated using the same procedure with Si replacing Si@rGO. Unless otherwise specified, the active-material loading was controlled at approximately 1.0 mg cm^−2^. Electrodes were punched into 12 mm disks and further vacuum-dried before transfer into the glovebox.

CR2025-type half batteries were assembled in the Ar glovebox using Si@rGO (or Si) as the working electrode, PEC-T as the solid electrolyte, and Li metal as the counter/reference electrode. To improve initial solid–solid contact without introducing excessive external pressure, the membrane/electrode stack was held at 60 °C under gentle uniaxial contact during assembly and then sealed in the coin-battery hardware. Stainless-steel (SS)/electrolyte/SS blocking cells were assembled for ionic-conductivity measurements, SS/electrolyte/Li cells for linear sweep voltammetry (LSV), and Li/electrolyte/Li cells for Li^+^-transference-number and plating/stripping measurements. The assembled batteries were equilibrated at the test temperature before measurement.

### 2.6. Materials Characterization

Phase structures were analyzed by X-ray diffraction (XRD, Rigaku Ultima IV, Tokyo, Japan) using Cu Kα radiation over 2θ = 10–70° at 10° min^−1^. Morphology and cross-sectional structure were characterized by field-emission scanning electron microscopy (FE-SEM, ZEISS Sigma 360, Carl Zeiss Microscopy GmbH, Jena, Germany) equipped with energy-dispersive X-ray spectroscopy (EDS). Transmission-electron microscopy and high-resolution imaging of Si@rGO were performed using an FEI Tecnai F20 TEM (FEI Company, Hillsboro, OR, USA). Differential scanning calorimetry (SC, DSC 214 Polyma, NETZSCH-Gerätebau GmbH, Selb, Germany) was conducted under N2 from −80 to 100 °C at 10 °C min^−1^. Thermogravimetric analysis was carried out under N_2_ using a TGA 550 analyzer (TA Instruments, New Castle, DE, USA) at 10 °C min^−1^ to 800 °C. Uniaxial tensile tests were performed using a UTM4103 universal testing machine (SUNS Technology Stock Co., Ltd., Shenzhen, China)at room temperature. Rectangular electrolyte specimens were cut to identical gauge dimensions before testing. Cross-sectional electrode thicknesses in the initial, lithiated and delithiated states were measured from SEM images obtained from separately recovered batteries at equivalent states of charge. Samples for ex situ SEM were handled to minimize air exposure.

### 2.7. Electrochemical Measurements

Electrochemical impedance spectroscopy (EIS) was performed with a CHI760F electrochemical workstation over 1 MHz to 1 Hz using a 5 mV alternating-current (AC) amplitude. The ionic conductivity (*σ*) of the PEC-T electrolyte was calculated according to Equation (1) [35]:

(1) $$\sigma =\frac{L}{\mathrm{RS}}$$

where L is membrane thickness, S is the contact area, and R is the bulk resistance obtained from the high-frequency intercept. Temperature-dependent conductivity was measured from 30 to 60 °C after equilibration at each temperature. The apparent activation energy was obtained by fitting the Arrhenius relationship between conductivity and reciprocal absolute temperature.

The apparent activation energy (Ea) for ionic transport was obtained by fitting the temperature-dependent ionic conductivity according to the Arrhenius relationship:

(2) $$\sigma (T)\;=\mathrm{Aexp}(-\frac{\mathrm{Ea}}{\mathrm{RT}})$$

where A is the pre-exponential factor, Ea is the apparent activation energy for ionic transport, R is the universal gas constant, and T is the absolute temperature.

The Li^+^ transference number (t_Li+_) was determined in Li/electrolyte/Li batteries using the Bruce-Vincent-Evans method. A direct-current (DC) polarization of ΔV = 10 mV was applied while recording the initial current I0 and steady-state current Iss; EIS spectra were collected immediately before and after polarization to obtain R0 and Rss. Oxidative stability was evaluated using SS/electrolyte/Li batteries by linear sweep voltammetry from 2 to 6 V at 1 mV s^−1^. The Li^+^ transference number was calculated using Equation (3) [36]:

(3) $$t_{\mathrm{Li}+}=\frac{I_{\mathrm{ss}}(\Delta V-I_{0}R_{0})}{I_{0}(\Delta V-I_{\mathrm{ss}}R_{\mathrm{ss}})}$$

Li/electrolyte/Li symmetric batteries were cycled galvanostatically using a LANHE battery-testing system. Step-current tests were performed sequentially at 0.02, 0.04, 0.06, 0.08 and 0.10 mA cm^−2^, while long-term stability was evaluated at 0.10 mA cm^−2^. Si@rGO/PEC-T/Li half batteries were tested between 0.01 and 1.50 V versus Li/Li^+^. Two formation cycles were first conducted at 0.1 C, followed by rate tests at 0.1, 0.2, 0.5, 1 and 2 C and a return to 0.1 C. Long-term cycling was performed at 1 C for 250 cycles. For consistency with the plotted data, 1 C was referenced to a nominal Si@rGO active-material capacity of 3.5 Ah g^−1^. Unless otherwise stated, galvanostatic solid-state battery tests were conducted at 60 °C. Specific capacities were calculated from the mass of Si@rGO active material. All key quantitative measurements were repeated using at least three independently prepared membranes or cells (n = 3). Quantitative data are reported as mean ± standard deviation (SD), while representative EIS and galvanostatic profiles are shown for clarity. The individual replicate values and the corresponding statistical results for the key physicochemical and electrochemical measurements are summarized in Table S2.

## 3. Results and Discussion

### 3.1. Structure and Morphology of Si@rGO and PEC-T

**Figure 2:**
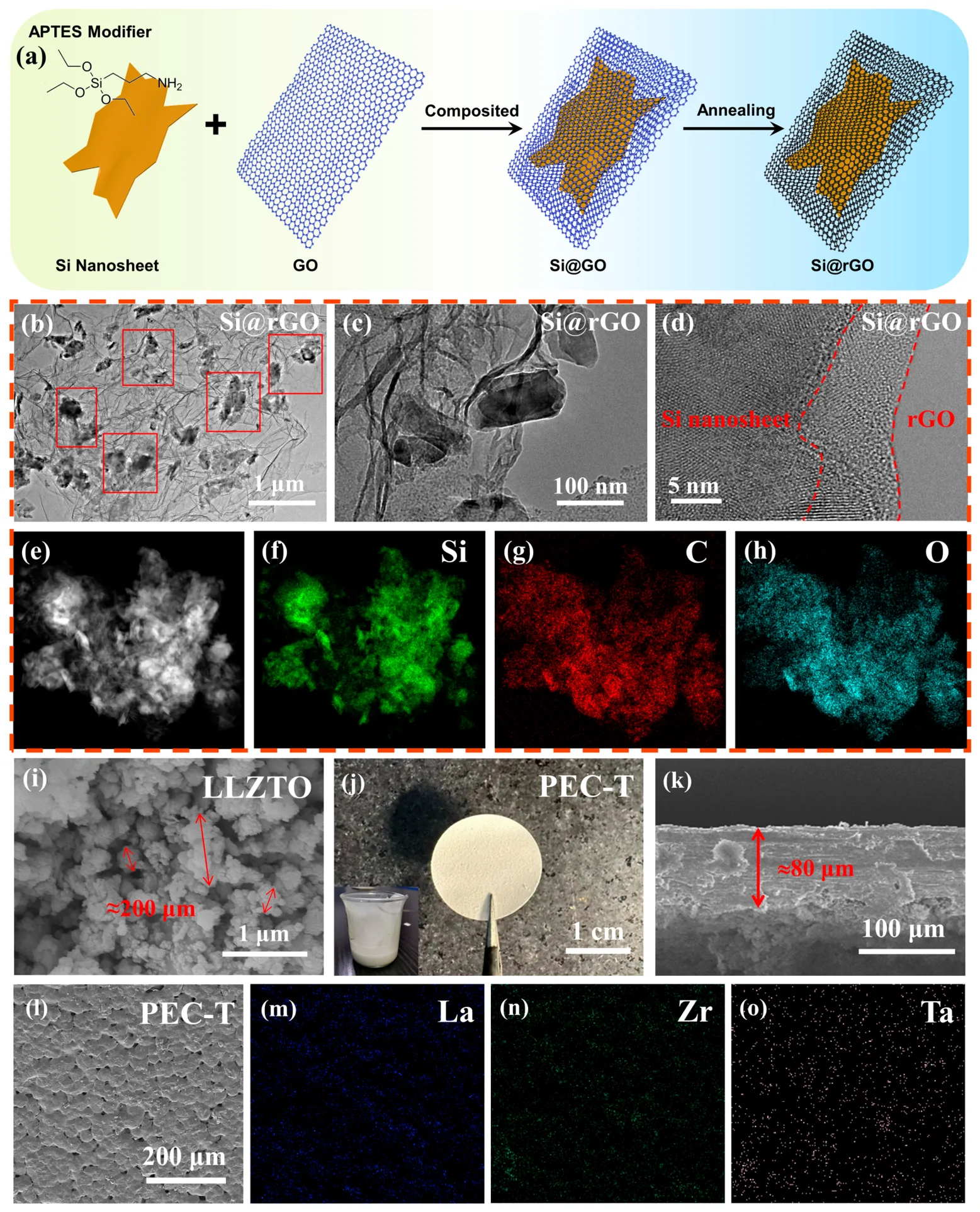
Microstructural characterization of the active materials and composite electrolyte. (**a**) Electrostatic assembly of APTES-modified Si nanosheets with GO followed by annealing to obtain Si@rGO. (**b**–**d**) TEM/HRTEM images of Si@rGO. (**e**–**h**) STEM image and corresponding Si, C, and O elemental maps of Si@rGO. (**i**) SEM image of the LLZTO filler showing an irregular submicron morphology. (**j**) Optical image of the free-standing PEC-T membrane. (**k**) Cross-sectional SEM image showing a membrane thickness of approximately 80 μm. (**l**) Surface SEM image of PEC-T. (**m**–**o**) Corresponding EDS elemental maps of La, Zr, and Ta in the PEC-T membrane, showing broadly distributed LLZTO-related elements within the observed region.

The Si@rGO composite is prepared through charge-directed assembly (Figure 2a). APTES functionalization introduces amino-containing surface groups on the Si nanosheets, making their surface sufficiently positive to interact electrostatically with negatively charged GO sheets in suspension. Subsequent annealing in Ar reduces GO to rGO while consolidating the interfacial contact. This route is advantageous over dry blending because the Si-rGO contact is established in the dispersed state before the carbon framework is thermally stabilized [37,38].

Transmission electron microscopy provides direct evidence of the resulting hybrid structure (Figure 2b–d). At the micrometer scale, the darker Si-containing domains are distributed within thin, wrinkled carbon sheets rather than forming a spatially isolated phase. Higher-magnification images reveal rGO intimately surrounding individual Si domains, and the 5 nm image resolves a sharp contrast transition between crystalline Si and the graphitic carbon region. STEM-EDS maps further show broadly overlapping Si and C distributions (Figure 2e–h), while the residual O signal is consistent with incomplete removal of oxygen-containing functionalities from rGO and/or native Si surface oxide. Such residual polar groups can be beneficial for interfacial adhesion but can also contribute to irreversible Li consumption during the first cycle; this trade-off becomes visible in the initial electrochemical profiles discussed later. A sample-specific oxygen percentage was not determined by XPS or bulk elemental analysis; therefore, the STEM-EDS O signal is interpreted qualitatively rather than as a quantitative oxygen measurement. For comparison, modified-Hummers GO has been reported to exhibit a C/O atomic ratio of 1.9 ± 0.1, whereas thermal reduction at 800 °C increases the C/O ratio to approximately 5.0 ± 0.7, demonstrating substantial but incomplete removal of oxygen-containing groups. These literature-reported values are provided only as chemical context and should not be interpreted as measurements of the present GO/rGO batch [39].

The LLZTO filler displays an irregular submicron morphology, with a characteristic particle dimension on the order of 200 nm in the representative SEM field (Figure 2i). More importantly, the elemental distribution within the final PEC-T membrane was directly examined by EDS mapping. As shown in Figure 2m–o, La, Zr, and Ta are distributed throughout the observed membrane region without obvious micron-scale elemental segregation, indicating that the Ta-doped LLZTO ceramic phase is effectively dispersed within the polymer matrix. The broadly overlapping distributions of La, Zr, and Ta further support the incorporation of LLZTO into the PEC-T membrane rather than the formation of isolated large ceramic-rich domains. In the final PEC-T film, the membrane can be handled as a free-standing disk (Figure 2j), has a cross-sectional thickness of approximately 80 μm (Figure 2k), and presents a continuous but microscopically textured surface (Figure 2l). The absence of large through-thickness cracks, together with the broadly distributed LLZTO-related elements, supports the formation of a structurally continuous polymer–ceramic composite membrane.

The membrane morphology also clarifies why a moderate ceramic loading is preferable here. At very high LLZTO fractions, particle-particle contacts may become more frequent, but the continuous deformable PEO phase is correspondingly diluted, which can reduce conformability and increase brittleness. At 10 wt% LLZTO, the microstructure remains polymer-continuous while incorporating sufficient inorganic filler to disturb PEO crystallization and reinforce the membrane. The La, Zr, and Ta elemental maps further indicate that the ceramic phase is not concentrated into obvious micron-scale segregated regions within the observed area, which is favorable for maintaining both ion-transport continuity and mechanical compliance [40,41].

### 3.2. LLZTO and Trace EC Cooperatively Regulate the Physicochemical State of PEO

**Figure 3:**
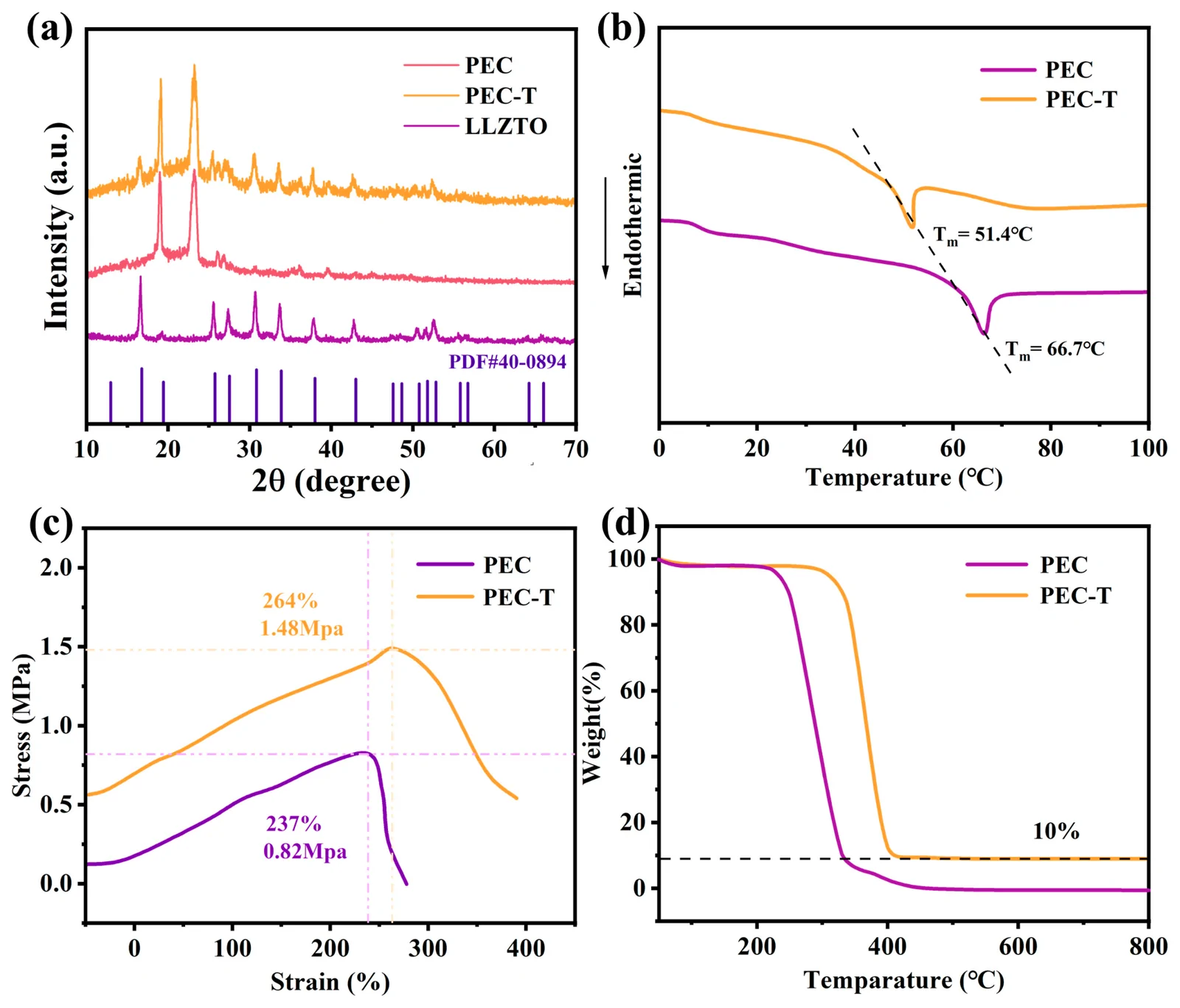
Physicochemical properties of PEC and PEC-T. (**a**) XRD patterns of PEC, PEC-T and LLZTO; the garnet reference pattern is shown at the bottom. (**b**) DSC curves showing a decrease in melting temperature from 66.7 °C for PEC to 51.4 °C for PEC-T. (**c**) Stress–strain curves: PEC reaches ~0.82 MPa and 237% strain, whereas PEC-T reaches ~1.48 MPa and 264% strain. (**d**) TGA profiles of PEC and PEC-T; the composite retains an inorganic residue of approximately 10% at high temperature.

The structural and thermal data in Figure 3 show that PEC-T is not simply PEC containing inert ceramic particles. X-ray diffraction of LLZTO is consistent with the cubic garnet phase, while the polymer-containing samples retain the characteristic PEO reflections. After addition of LLZTO, the relative intensity and definition of the PEO-related peaks decrease, indicating disruption of long-range chain packing. This is the structural signature expected when ceramic surfaces interfere with regular PEO lamellae and create a larger amorphous interfacial fraction [42,43]. Because Li^+^ transport in PEO is coupled to local segmental motion, this reduction in crystalline order is directly relevant to ion mobility rather than being a purely structural observation.

Differential scanning calorimetry provides a quantitative measure of this change. PEC melts at 66.7 °C, whereas the melting endotherm of PEC-T shifts to 51.4 °C (Figure 3b). The 15.3 °C decrease demonstrates a substantial reduction in the thermal stability of ordered PEO domains. EC contributes plasticization and salt dissociation, while LLZTO produces a high-area polymer/ceramic boundary that frustrates recrystallization. Their combined action therefore creates a broader temperature range in which chain segments are mobile enough to support Li^+^ transport. The strong conductivity increase with temperature shown later is consistent with this thermally activated segmental contribution.

Importantly, the reduction in crystallinity does not cause a loss of mechanical integrity. PEC exhibits a tensile strength of approximately 0.82 MPa and an elongation at break of 237%, whereas PEC-T reaches 1.48 MPa and 264%, respectively (Figure 3c). Simultaneous improvement of strength and extensibility is particularly valuable for a silicon-facing electrolyte: strength helps resist localized penetration and stress concentration, while high strain tolerance enables the membrane to follow gradual electrode displacement without brittle fracture. The ceramic filler therefore acts as a reinforcing phase without eliminating the characteristic compliance of PEO [44].

Thermogravimetric analysis further reveals improved high-temperature dimensional/chemical stability of the composite. PEC loses most of its mass over the polymer decomposition regime, while PEC-T exhibits a delayed main mass-loss process and leaves an approximately 10% high-temperature residue (Figure 3d), consistent with the deliberately introduced inorganic fraction. The combined DSC, tensile, and TGA results define the central materials trade-off achieved by PEC-T: the polymer is rendered less crystalline and more mobile at the molecular scale, yet the macroscopic membrane becomes mechanically stronger and thermally more robust.

### 3.3. Ion Transport, Electrochemical Stability, and Li-Interface Durability

**Figure 4:**
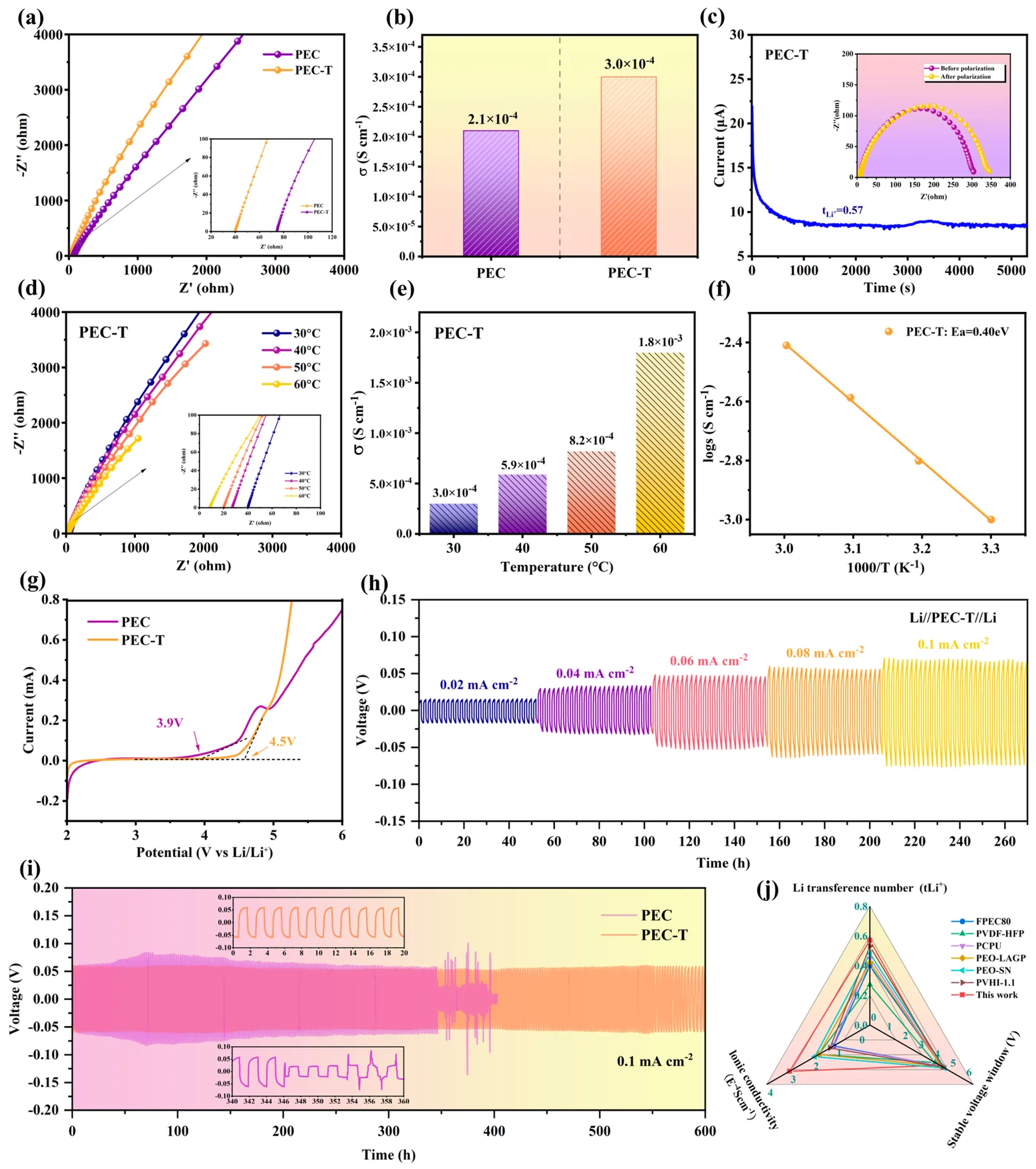
Electrochemical transport and interfacial stability of PEC-T. (**a**) Nyquist plots of PEC and PEC-T. (**b**) Ionic conductivities of PEC (2.1 × 10^−4^ S cm^−1^) and PEC-T (3.0 × 10^−4^ S cm^−1^) at the baseline condition. (**c**) DC polarization of Li/PEC-T/Li and EIS before/after polarization, giving t_Li+_ = 0.57. (**d**,**e**) Temperature-dependent EIS and ionic conductivity of PEC-T from 30 to 60 °C. (**f**) Arrhenius analysis yielding an apparent activation energy of 0.40 eV. (**g**) LSV curves showing oxidation onset near 3.9 V for PEC and ~4.5 V for PEC-T. (**h**) Li/PEC-T/Li cycling under stepwise current densities from 0.02 to 0.10 mA cm^−2^. (**i**) Long-term Li symmetric-battery cycling at 0.10 mA cm^−2^. (**j**) Literature-based radar comparison of ionic conductivity, Li^+^ transference number, and oxidative stability. The numerical values, measurement temperatures, and corresponding data sources are summarized in Table S3.

The transport benefit of the composite formulation is apparent from the blocking-electrode impedance spectra (Figure 4a). PEC-T exhibits a smaller high-frequency resistance than PEC, corresponding to an ionic conductivity increase from 2.1 × 10^−4^ to 3.0 × 10^−4^ S cm^−1^ under the baseline measurement condition (30 °C). The absolute gain is modest compared with the orders-of-magnitude conductivity change that occurs when PEO crosses its melting regime, but it is mechanistically significant because it is obtained while the membrane also becomes stronger. The increase is consistent with two parallel effects: a larger amorphous PEO fraction and additional ion-conduction environments at the PEO/LiTFSI/LLZTO interfaces [45].

Temperature-dependent measurements show that PEC-T reaches 5.9 × 10^−4^, 8.2 × 10^−4^, and 1.8 × 10^−3^ S cm−^1^ at 40, 50, and 60 °C, respectively (Figure 4d,e). The Arrhenius representation gives an apparent activation energy of 0.40 eV (Figure 4f). The obtained Ea of 0.40 eV represents the apparent transport barrier over the investigated temperature range of 30–60 °C. Its magnitude is consistent with the strongly temperature-dependent segmental dynamics of the PEO-rich matrix, indicating that LLZTO enhances transport without replacing polymer-chain motion as the dominant kinetic contribution. The strong temperature dependence is expected for PEO-rich electrolytes and indicates that polymer segmental dynamics remain an important part of the transport mechanism even after LLZTO addition. In other words, LLZTO acts as an active modifier of a polymer-continuous electrolyte, not as a fully percolating ceramic skeleton. This distinction is important when interpreting the excellent flexibility of the membrane and its ability to maintain contact with Si.

A second relevant transport parameter is the cation transference number. Direct-current polarization of Li/PEC-T/Li combined with impedance measurements before and after polarization yields t_Li+_ = 0.57 (Figure 4c), substantially above the values typical of unmodified PEO-LiTFSI electrolytes in which anion motion contributes strongly to the total ionic current [43]. A higher t_Li+_ reduces the growth of salt concentration gradients under current and is therefore beneficial for both polarization control and homogeneous Li-ion flux at the buried interface.

The composite also improves oxidative tolerance. In linear sweep voltammetry, PEC shows a pronounced oxidation rise at approximately 3.9 V, whereas PEC-T shifts the onset to ~4.5 V (Figure 4g). The result does not imply that every electrolyte component is thermodynamically stable to 4.5 V; rather, it reflects the effective kinetic stability of the composite under the measurement protocol. LLZTO and the modified polymer coordination environment can reduce the density of easily oxidized local configurations, while the small EC fraction avoids the large free-solvent inventory characteristic of gel electrolytes.

Lithium symmetric batteries provide a stringent probe of the combined transport and mechanical behavior. When the current density is stepped from 0.02 to 0.10 mA cm^−2^, Li/PEC-T/Li exhibits regular polarization without abrupt voltage collapse over approximately 260 h (Figure 4h). More notably, at 0.10 mA cm^−2^ the PEC-T battery maintains stable plating/stripping for 600 h, whereas the PEC battery develops severe voltage instability after roughly 350–380 h (Figure 4i). The long-term improvement is consistent with a membrane that simultaneously lowers ionic resistance, redistributes current more uniformly, and better resists mechanically localized penetration. These roles are mutually reinforcing: more uniform ion flux reduces the probability of a local high-current defect, while higher mechanical strength slows the conversion of such a defect into a short-circuit pathway [46]. As shown in Figure 4j, PEC-T exhibits favorable overall electrochemical performance compared with representative polymer-based and composite solid electrolytes reported in the literature [47,48,49,50,51,52]. In particular, PEC-T combines a relatively high ionic conductivity of 3.0 × 10^−4^ S cm^−1^ at 30 °C, a Li^+^ transference number of 0.57, and an oxidative stability of approximately 4.5 V. The balanced performance across these key parameters demonstrates the good ion-transport capability and electrochemical stability of the PEC-T electrolyte, supporting its suitability for solid-state lithium batteries.

### 3.4. rGO Confinement Reduces Irreversible Dimensional Evolution of the Si Electrode

**Figure 5:**
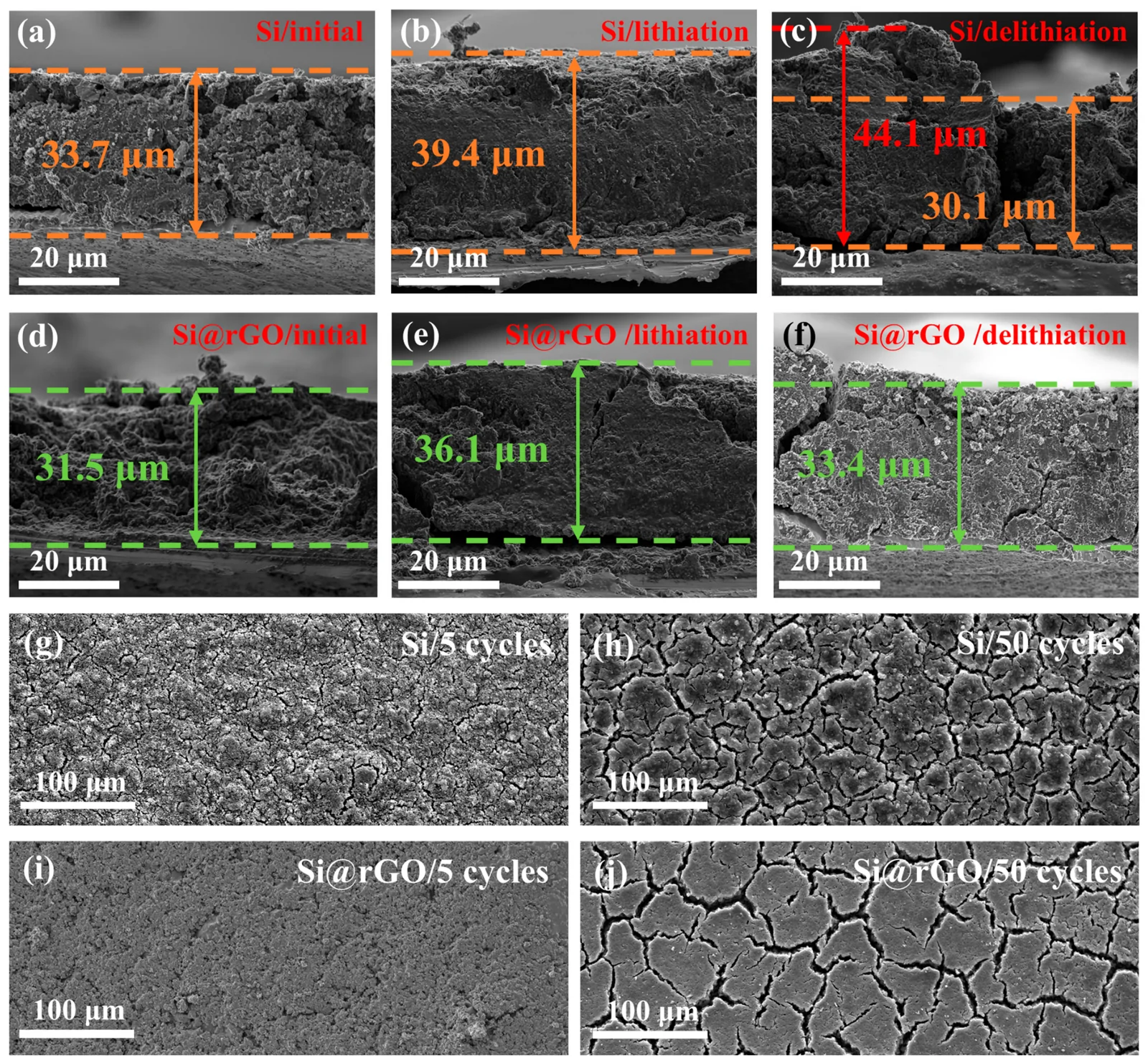
Chemo-mechanical evolution of Si and Si@rGO electrodes. Cross-sectional SEM images of bare Si in the (**a**) initial, (**b**) lithiated and (**c**) delithiated states, and Si@rGO in the (**d**) initial, (**e**) lithiated and (**f**) delithiated states. Top-view SEM images of bare Si after (**g**) 5 and (**h**) 50 cycles and Si@rGO after (**i**) 5 and (**j**) 50 cycles. The rGO framework reduces irreversible electrode-thickness evolution and moderates, but does not completely eliminate, cycling-induced cracking.

The central mechanical hypothesis of the electrode design can be tested directly by comparing cross-sectional thickness changes of bare Si and Si@rGO electrodes (Figure 5). The pristine Si electrode is ~33.7 μm thick and increases to ~39.4 μm after lithiation, corresponding to an apparent electrode-level thickness increase of ~17%. Although this value is far below the intrinsic volumetric expansion of fully lithiated crystalline Si, the discrepancy is expected because composite electrodes contain pores, binder, conductive additive, and nonuniformly lithiated domains. More important than the absolute value is what occurs after delithiation: the Si electrode develops pronounced local height variations, with a protruding region reaching ~44.1 μm while a lower compacted region is ~30.1 μm. This spatially heterogeneous residual deformation is a direct precursor to interfacial contact loss in a solid-state architecture.

Si@rGO shows a different evolution. Its initial thickness is ~31.5 μm and increases to ~36.1 μm after lithiation (~15% apparent expansion). After delithiation, the thickness relaxes to ~33.4 μm, leaving a much smaller net dimensional offset from the initial state. The rGO scaffold cannot eliminate Si alloying strain, but it can redistribute local displacement, maintain electronic contact across partially separated Si domains, and provide compliant free volume at the nanoscale. The result is a more reversible electrode-scale breathing mode rather than strongly localized residual deformation [53,54].

The SEM observations after repeated cycling further reveal the structural advantage of the Si@rGO composite. Although surface cracks are still observed after prolonged cycling, the Si@rGO electrode exhibits a markedly flatter and more homogeneous surface than the bare Si electrode, with reduced localized deformation and less severe morphological disruption. This improvement is attributed to the intimate coupling between Si and the flexible rGO framework, which redistributes the strain generated during repeated lithiation/delithiation and constrains excessive local expansion of Si. As a result, the dimensional evolution of the composite electrode becomes more spatially uniform rather than being concentrated at isolated regions. Such morphological homogenization is particularly important in solid-state batteries, where local cracking and surface roughening can directly deteriorate the buried electrode/electrolyte contact and interrupt continuous Li^+^ transport pathways. Therefore, the role of rGO is not to completely eliminate Si fracture, but to regulate its mechanical evolution toward a smoother and more uniform electrode surface, thereby preserving more stable interfacial contact during extended cycling.

The electrolyte and electrode thus solve complementary parts of the same contact problem. Si@rGO makes the moving boundary more spatially uniform, while PEC-T makes the opposing boundary more compliant and mechanically resilient. The electrochemical response of the complete battery should therefore exhibit less progressive polarization than would be expected from either bare Si or a more crystalline PEO electrolyte.

### 3.5. Electrochemical Performance of Si@rGO/PEC-T/Li

**Figure 6:**
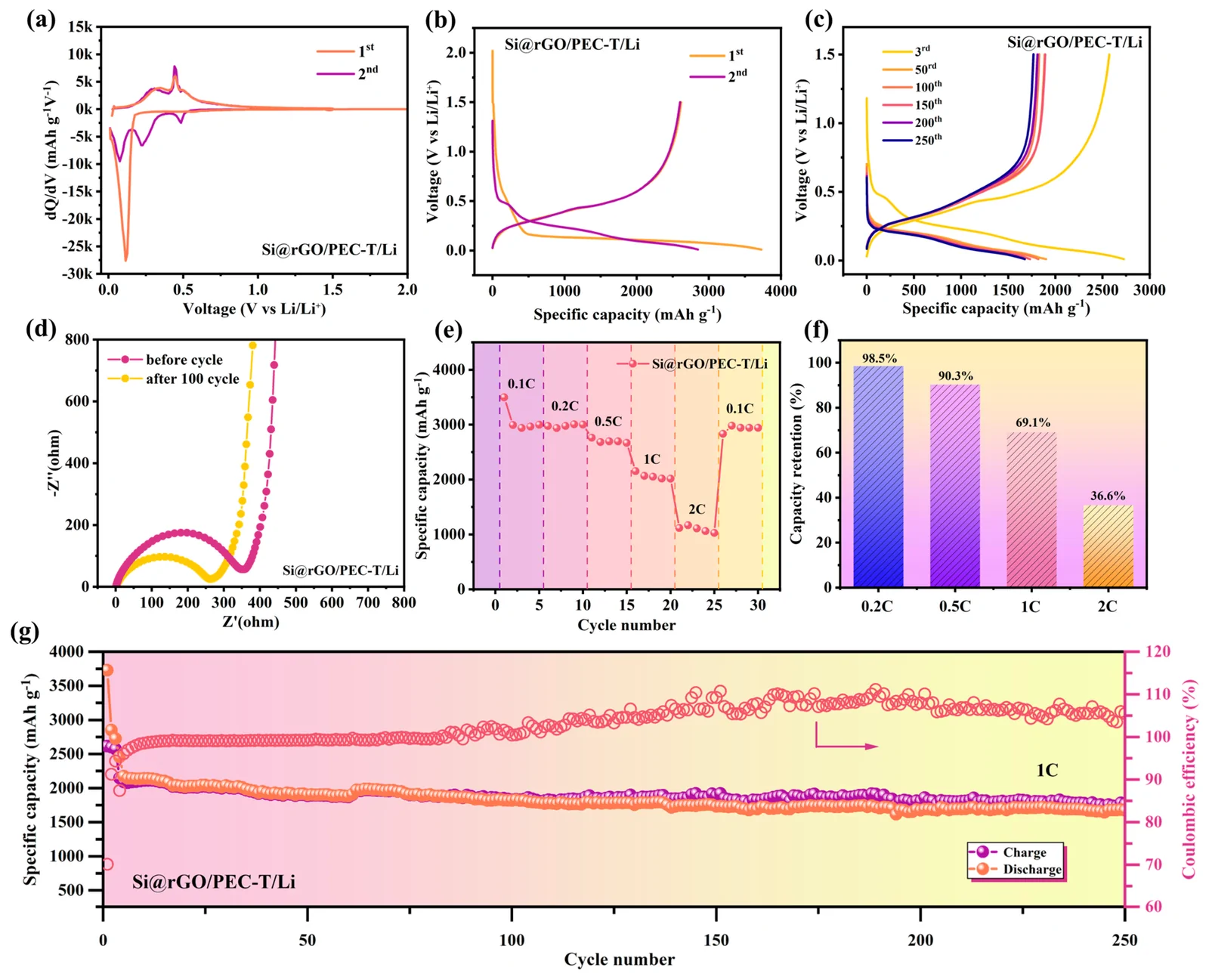
Electrochemical performance of Si@rGO/PEC-T/Li batteries. (**a**) Differential-capacity curves for the first two cycles. (**b**) First and second galvanostatic voltage profiles. (**c**) Representative voltage profiles from the 3rd to 250th cycle. (**d**) EIS spectra before cycling and after 100 cycles. (**e**) Rate capability from 0.1 to 2 C and recovery at 0.1 C. (**f**) Capacity retention at 0.2, 0.5, 1 and 2 C. (**g**) Long-term charge/discharge capacity and Coulombic efficiency over 250 cycles at 1 C.

The first two cycles of the Si@rGO/PEC-T/Li battery display the electrochemical fingerprints expected for Si alloying (Figure 6a,b). During the first lithiation, the differential-capacity profile contains a strong low-voltage reduction feature associated with conversion of crystalline Si toward amorphous Li_x_Si together with formation of the initial interphase. On delithiation, broad oxidation features appear in the ~0.3–0.5 V region, consistent with dealloying of amorphous Li_x_Si. The large first-cycle irreversible capacity visible in the galvanostatic profiles is therefore attributable to a combination of interphase formation, residual oxygen-containing groups on rGO/SiO_x_, and incomplete recovery of lithium from newly formed amorphous domains. From the second cycle onward, the profiles become substantially more reproducible, indicating that the dominant irreversible reconstruction occurs during formation rather than continuing unchecked.

Long-term voltage profiles further support stabilization of the reaction pathway (Figure 6c). After the early conditioning cycles, the 50th through 250th profiles retain a similar shape, with only a gradual reduction in accessible capacity. This overlap is notable for a high-capacity Si electrode in a solid-state configuration because electrochemically isolated regions would otherwise appear as increasing polarization and progressive loss of plateau/sloping capacity. The impedance spectra measured before cycling and after 100 cycles show a smaller dominant semicircle after cycling (Figure 6d), suggesting interfacial activation and improved effective contact rather than monotonic resistance growth. A reasonable interpretation is that early cycling allows the compliant PEC-T phase to mechanically settle against the rough Si@rGO surface while the interphase reaches a more stable configuration.

The rate response demonstrates that the high capacity is not limited to near-equilibrium cycling (Figure 6e). The battery delivers approximately 3.0 Ah g^−1^ in the low-rate regime, retains substantial capacity as the rate is increased through 0.2, 0.5 and 1 C, and still provides ~1.1 Ah g^−1^ at 2 C. When the rate is returned to 0.1 C, the capacity recovers to nearly 3.0 Ah g^−1^, indicating that the temporary loss at high rate is predominantly kinetic rather than caused by irreversible structural collapse. Quantitatively, the capacity retention values relative to the low-rate reference are 98.5% at 0.2 C, 90.3% at 0.5 C, 69.1% at 1 C and 36.6% at 2 C (Figure 6f). The high-rate limitation is consistent with the 0.40 eV apparent transport barrier and the fact that PEC-T remains a polymer-continuous electrolyte rather than a fully ceramic percolating network.

Cycling at 1 C provides the most stringent system-level test (Figure 6g). After initial formation and relaxation, the charge and discharge capacities stabilize near the 2 Ah g^−1^ level and decay gradually rather than catastrophically, reaching approximately 1.8 Ah g^−1^ after 250 cycles. After the early formation cycles, the apparent Coulombic efficiency remains close to unity, with occasional slight excursions above 100%. Such values should not be interpreted as a true faradaic efficiency greater than unity. In Si electrodes, a small amount of Li that remains kinetically trapped during preceding cycles may be released during subsequent delithiation as the electrode/interphase gradually relaxes. In addition, small baseline and capacity-integration offsets can produce a slight imbalance between lithiation and delithiation capacities. Therefore, the apparent values slightly above 100% are attributed to Li-inventory redistribution together with measurement/integration uncertainty rather than to net generation of cyclable Li. The remaining capacity fade is consistent with the residual cracking observed by SEM and with unavoidable interphase evolution at highly lithiated Si. Nevertheless, the absence of abrupt failure demonstrates that neither crack formation nor Si breathing necessarily leads to immediate solid–solid contact loss when both sides of the interface are designed to accommodate displacement.

To further evaluate the electrochemical performance of the present Si@rGO/PEC-T electrode, a quantitative comparison with representative Si-based anodes reported in the literature is summarized in Table S4. The present electrode exhibits competitive cycling stability and rate capability, retaining approximately 1.8 Ah g^−1^ after 250 cycles at 1 C and delivering approximately 1.1 Ah g^−1^ at 2 C. These results demonstrate the favorable electrochemical performance of the Si@rGO/PEC-T configuration among representative Si-based anode systems.

Taken together, Figure 3, Figure 4, Figure 5 and Figure 6 support a three-level mechanism. At the molecular level, EC and LLZTO reduce PEO ordering and improve Li^+^ mobility. At the membrane level, LLZTO raises strength while preserving high extensibility, enabling long-term Li-interface stability. At the electrode level, rGO redistributes Si deformation and maintains electronic continuity. The electrochemical advantage arises from the alignment of all three length scales: faster ion supply reduces concentration polarization, a tougher yet compliant electrolyte preserves contact, and a more reversible electrode morphology lowers the amplitude of interfacial displacement that the electrolyte must accommodate. This coupled mechanism provides a generalizable design rule for high-expansion alloy anodes in solid-state batteries.

## 4. Conclusions

A contact-stable solid-state silicon battery was developed by co-engineering the active electrode and the adjacent polymer–ceramic electrolyte. Electrostatic assembly and thermal reduction created Si@rGO in which flexible conductive graphene sheets constrain nonuniform Si displacement and preserve electronic connectivity. In parallel, the PEC-T electrolyte combines PEO-LiTFSI with a small EC fraction and 10 wt% LLZTO. The formulation lowers the PEO melting temperature from 66.7 to 51.4 °C, raises tensile strength from 0.82 to 1.48 MPa while increasing elongation from 237% to 264%, and provides 3.0 × 10^−4^ S cm^−1^ ionic conductivity at 30 °C, t_Li+_ = 0.57, and an effective oxidative stability of ~4.5 V. The improved transport and mechanics support stable Li plating/stripping for 600 h at 0.1 mA cm^−2^. At the Si interface, rGO reduces irreversible electrode-thickness evolution, and the compliant PEC-T membrane remains capable of following the residual breathing. The resulting Si@rGO/PEC-T/Li battery shows high reversible capacity, strong rate recovery, and approximately 1.7–1.8 Ah g^−1^ after 250 cycles at 1 C. More broadly, the work demonstrates that high-capacity solid-state alloy anodes should be designed through coupled control of ion solvation, polymer crystallinity, composite mechanics, and electrode strain accommodation. Optimizing only the intrinsic conductivity of the electrolyte or only the nanostructure of Si leaves the buried interface as the dominant failure point; co-designing both sides of that interface converts large Si volume change from a catastrophic contact-loss mechanism into a manageable, reversible deformation mode.

## Data Availability

The original contributions presented in this study are included in the article/Supplementary Material. Further inquiries can be directed to the corresponding authors.

## Supplementary Materials

The following supporting information can be downloaded at: https://www.mdpi.com/article/10.3390/polym18192374/s1.

## Abbreviations

Materials and Electrolyte Formulations	
Abbreviation	Definition
APTES	3-Aminopropyltriethoxysilane
CMC	Carboxymethyl cellulose
EC	Ethylene carbonate
GO	Graphene oxide
LiTFSI	Lithium bis(trifluoromethanesulfonyl)imide
LLZTO	Ta-doped lithium lanthanum zirconium oxide (Li_6.5_La_3_Zr_1.5_Ta_0.5_O_12_)
PEC	PEO–LiTFSI–EC electrolyte
PEC-T	LLZTO-reinforced PEO–LiTFSI–EC electrolyte
PEO	Poly(ethylene oxide)
rGO	Reduced graphene oxide
Si@rGO	Silicon/reduced graphene oxide composite
Structural and Physicochemical Characterization	
Abbreviation	Definition
DSC	Differential scanning calorimetry
EDS	Energy-dispersive X-ray spectroscopy
FE-SEM	Field-emission scanning electron microscopy
HRTEM	High-resolution transmission electron microscopy
SEM	Scanning electron microscopy
STEM	Scanning transmission electron microscopy
TEM	Transmission electron microscopy
TGA	Thermogravimetric analysis
XRD	X-ray diffraction
