# Supplementary material for: Graphene-Confined Silicon and a Li6.5La3Zr1.5Ta0.5O12-Reinforced Plasticized Poly(ethylene oxide) Electrolyte Enable Contact-Stable Solid-State Lithium Batteries
Source: Polymers (Basel). 2026 Sep 29;18(19):2374. doi: 10.3390/polym18192374 (PMC13666941; doi:10.3390/polym18192374)
Supplement: Supplementary file 1 [file polymers-18-02374-s001.zip › polymers-4559977-supplementary.pdf]

Supporting Information

Xianzheng Liu [1,2], Nashrah Hani Jamadon [2,*], Jiayi Li [1], Xiaoxi Liu [3], Wenbo Jia [1], Rongji Tang [2], Liancheng Zheng [2], Zhenhua Liu [1] and Dongpo Wei [1,*]

[1] College of Mechanical Engineering, Shandong Huayu University of Technology, Dezhou 253034, China; lxz@huayu.edu.cn (X.L.); lijiayi19952565@163.com (J.L.); 17745107275@163.com (W.J.); liuzh@huayu.edu.cn (Z.L.)

[2] Department of Mechanical and Manufacturing Engineering, Faculty of Engineering and Built Environment, Universiti Kebangsaan Malaysia, Bangi 43600, Selangor, Malaysia; p159500@siswa.ukm.edu.my (R.T.); p159501@siswa.ukm.edu.my (L.Z.)

[3] Department of Electrical, Electronic and Systems Engineering, Faculty of Engineering and Built Environment, Universiti Kebangsaan Malaysia, Bangi 43600, Selangor, Malaysia; p159656@siswa.ukm.edu.my

* Correspondence: nashrahhani@ukm.edu.my (N.H.J.); wei31028@yeah.net (D.W.)

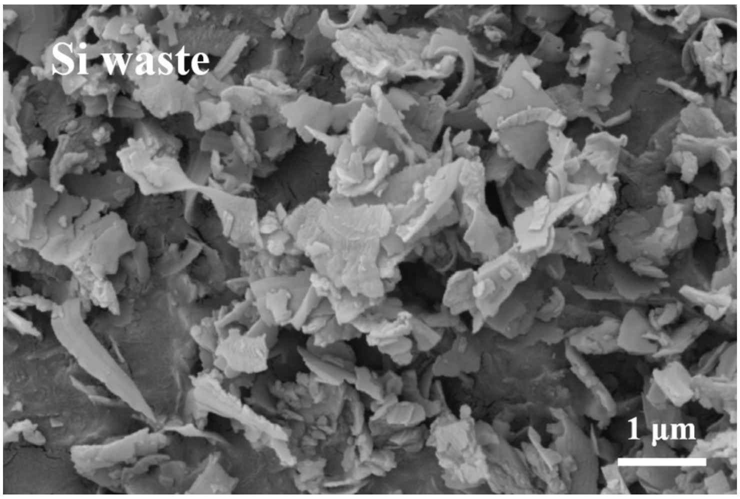

Figure S1. SEM micrograph of the high-temperature-pretreated photovoltaic wafer-cutting silicon waste used as the Si precursor. The particles exhibit an irregular flake-like and fractured morphology, with apparent lateral dimensions mainly in the ~0.5–3 μm range and occasional larger or overlapping features approaching ~5 μm. The dimensions were estimated directly from representative SEM images and should not be interpreted as a laser-diffraction D50 value.

Table S1. Bulk elemental composition of the high-temperature-pretreated photovoltaic wafer-cutting silicon waste used in this work.

Element	Content (wt%)	Analytical method
Si	91.70	ICP-OES/XRF
O	7.40	Oxygen analysis
Fe	0.28	ICP-OES
Al	0.19	ICP-OES
C	0.16	Carbon analyzer
Ca	0.07	ICP-OES
Other detectable impurities	0.20	ICP-OES
**Total**	**100.00**	—

Table S2. Reproducibility of the key physicochemical and electrochemical properties obtained from three independently prepared samples or cells.

Property	Sample	Replicate 1	Replicate 2	Replicate 3	Mean ± SD
Tensile strength (MPa)	PEC	0.79	0.83	0.84	0.82 ± 0.03
Tensile strength (MPa)	PEC-T	1.43	1.49	1.52	1.48 ± 0.05
Elongation at break (%)	PEC	232	239	240	237 ± 4
Elongation at break (%)	PEC-T	258	266	268	264 ± 5
Ionic conductivity at 30 °C ($\times 10^{-4}$ S cm^{-1})	PEC	2.04	2.12	2.14	2.10 ± 0.05
Ionic conductivity at 30 °C ($\times 10^{-4}$ S cm^{-1})	PEC-T	2.94	3.02	3.04	3.00 ± 0.05
Ionic conductivity at 40 °C ($\times 10^{-4}$ S cm^{-1})	PEC-T	5.72	5.93	6.05	5.90 ± 0.17
Ionic conductivity at 50 °C ($\times 10^{-4}$ S cm^{-1})	PEC-T	7.95	8.21	8.44	8.20 ± 0.25
Ionic conductivity at 60 °C ($\times 10^{-3}$ S cm^{-1})	PEC-T	1.74	1.81	1.85	1.80 ± 0.06
Li^+ transference number	PEC-T	0.55	0.57	0.59	0.57 ± 0.02
Capacity after 250 cycles at 1 C (Ah g^{-1})	Si@rGO/PEC-T/Li	1.76	1.82	1.79	1.79 ± 0.03

Table S3. Numerical values used for the literature-based radar comparison in Figure 4j.

**Electrolyte**	**Ionic conductivity ($S\ cm^{-1}$)**	**Test temperature**	**Stable voltage window (V)**	**Li^+ transference number**	**Ref.**
FPEC80	1.08×10^{-4}	30 °C	4.5	0.4	[1]
PVDF-HFP	1.73×10^{-5}	Not specified in Table S3	4.1	0.27	[2]
PCPU	1.12×10^{-4}	80 °C	4.5	0.45 (80 °C)	[3]
PEO-LAGP	1.73×10^{-4}	25 °C	4.5	0.5	[4]
PEO-SN	1.90×10^{-4}	25 °C	4.7	0.48 (25 °C)	[5]
PVHLi-1.1	1.24×10^{-4}	25 °C	4.7	0.53 (25 °C)	[6]
PEC-T, this work	3.0×10^{-4}	30 °C	~4.5	0.57	This work

Table S4. Quantitative comparison of the electrochemical performance of the present Si@rGO/PEC-T electrode with representative Si-based anodes reported in the literature.

Si-based anode	Cell/system	Test condition	Quantitative performance	Ref.
**Si@rGO/PEC-T, this work**	Polymer–ceramic solid-state half-cell	60 °C; loading ~1.0 mg cm^{-2}	~1.8 Ah g^{-1} after 250 cycles at 1 C; ~1.1 Ah g^{-1} at 2 C	This work
Si/LPSCl composite	Sulfide solid-state half-cell	0.1 C; 50 MPa	21.9% capacity retention after 100 cycles	[12]
SE-free Si	Sulfide solid-state half-cell	0.1 C; 50 MPa	29.3% capacity retention after 100 cycles	[12]
$Si/Li_{21}Si_5$	All-solid-state battery	Reported conditions	ICE = 97.8%; areal capacity = 17.9 mAh cm^{-2}; expansion = 18.9%	[10]
Graphene-scaffolded Si/graphite	Liquid-electrolyte LIB half-cell	75–372 mA g^{-1}	559 mAh g^{-1} at 75 mA g^{-1}; ~90% retention after 100 cycles at 372 mA g^{-1}	[48]

References

1. Zhao, Y.; Bai, Y.; Bai, Y.; An, M.; Chen, G.; Li, W.; Li, C.; Zhou, Y. A rational design of solid polymer electrolyte with high salt concentration for lithium battery. *Journal of Power Sources* **2018**, *407*, 23-30.
2. Sun, J.; Yao, X.; Li, Y.; Zhang, Q.; Hou, C.; Shi, Q.; Wang, H. Composite Solid Electrolytes: Facilitating Interfacial Stability Via Bilayer Heterostructure Solid Electrolyte Toward High-energy, Safe and Adaptable Lithium Batteries (Adv. Energy Mater. 31/2020). *Advanced Energy Materials* **2020**, *10*, 2070131.
3. Bao, J.; Shi, G.; Tao, C.; Wang, C.; Zhu, C.; Cheng, L.; Qian, G.; Chen, C. Polycarbonate-based polyurethane as a polymer electrolyte matrix for all-solid-state lithium batteries. *Journal of Power Sources* **2018**, *389*, 84-92.
4. Liu, M.; Cheng, Z.; Ganapathy, S.; Wang, C.; Haverkate, L.A.; Tułodziecki, M.; Unnikrishnan, S.; Wagemaker, M. Tandem interface and bulk Li-ion transport in a hybrid solid electrolyte with microsized active filler. *ACS Energy Letters* **2019**, *4*, 2336-2342.
5. Xu, S.; Sun, Z.; Sun, C.; Li, F.; Chen, K.; Zhang, Z.; Hou, G.; Cheng, H.M.; Li, F. Homogeneous and fast ion conduction of PEO-based solid-state electrolyte at low temperature. *Advanced Functional Materials* **2020**, *30*, 2007172.
6. Liu, W.; Yi, C.; Li, L.; Liu, S.; Gui, Q.; Ba, D.; Li, Y.; Peng, D.; Liu, J. Designing polymer-in-salt electrolyte and fully infiltrated 3D electrode for integrated solid-state lithium batteries. *Angewandte Chemie International Edition* **2021**, *60*, 12931-12940.
